# Aberrant splicing caused by exonic single nucleotide variants positioned 2nd or 3rd to the last nucleotide in the *COL4A5* gene

**DOI:** 10.1007/s10157-022-02294-x

**Published:** 2022-11-12

**Authors:** Eri Okada, Yuya Aoto, Tomoko Horinouchi, Tomohiko Yamamura, Yuta Ichikawa, Yu Tanaka, Chika Ueda, Hideaki Kitakado, Atsushi Kondo, Nana Sakakibara, Ryota Suzuki, Joichi Usui, Kunihiro Yamagata, Kazumoto Iijima, Kandai Nozu

**Affiliations:** 1 Department of Pediatrics, Kobe University Graduate School of Medicine, 7-5-1 Kusunoki-Cho, Chuo-Ku, Kobe, Hyogo 650-0017 Japan; 2 Department of Nephrology, Faculty of Medicine, University of Tsukuba, Tsukuba, Japan; 3 Department of Pediatrics, Hokkaido University Graduate School of Medicine, Sapporo, Japan; 4 Hyogo Prefectural Kobe Children’s Hospital, Kobe, Japan; 5 Department of Advanced Pediatric Medicine, Kobe University Graduate School of Medicine, Kobe, Japan

**Keywords:** COL4A5, Alport syndrome, Minigene assay, Aberrant splicing, Single nucleotide substitution, Inherited kidney disease

## Abstract

**Background and objectives:**

The evident genotype–phenotype correlation shown by the X-linked Alport syndrome warrants the assessment of the impact of identified gene variants on aberrant splicing. We previously reported that single nucleotide variants (SNVs) in the last nucleotide of exons in *COL4A5* cause aberrant splicing. It is known that the nucleotides located 2nd and 3rd to the last nucleotides of exons can also play an essential role in the first step of the splicing process. In this study, we aimed to investigate whether SNVs positioned 2nd or 3rd to the last nucleotide of exons in *COL4A5* resulted in aberrant splicing.

**Methods:**

We selected eight candidate variants: six from the Human Gene Variant Database Professional and two from our cohort. We performed an *in-vitro* splicing assay and reverse transcription-polymerase chain reaction (RT-PCR) for messenger RNA obtained from patients, if available.

**Results:**

The candidate variants were initially classified into the following groups: three nonsense, two missense, and three synonymous variants. Splicing assays and RT-PCR for messenger RNA revealed that six of the eight variants caused aberrant splicing. Four variants, initially classified as non-truncating variants, were found to be truncating ones, which usually show relatively more severe phenotypes.

**Conclusion:**

We revealed that exonic SNVs positioned 2nd or 3rd to the last nucleotide of exons in the *COL4A5* were responsible for aberrant splicing. The results of our study suggest that attention should be paid when interpreting the pathogenicity of exonic SNVs near the 5′ splice site.

**Supplementary Information:**

The online version contains supplementary material available at 10.1007/s10157-022-02294-x.

## Introduction

Alport syndrome (AS) is an inherited renal disease characterized by hematuria, proteinuria, progressive kidney dysfunction, and extrarenal manifestations such as hearing loss and ocular abnormalities. AS is caused by variants in the *COL4A3* (OMIM, # 120,070), *COL4A4* (OMIM, # 120,131), and *COL4A5* (OMIM, # 303,630) genes encoding type IV collagen α3, α4 and α5 chains, respectively, which are the major components of the glomerular basement membrane [1–3]. Families with AS show X-linked, autosomal recessive, or autosomal dominant inheritance patterns. Among them, X-linked AS (XLAS) is predominant and is caused by rare variants of *COL4A5* on chromosome Xq22.

It has been reported that male patients with XLAS show significant genotype–phenotype correlations; patients with nonsense variants show the most severe phenotype, those harboring missense variants show a mild phenotype and those with splicing variants present a moderate phenotype [4–8]. Moreover, among the splicing variants, in-frame variants show significantly milder phenotypes compared to frameshift variants [7–11]. Therefore, it is crucial to correctly interpret the pathogenicity of identified variants. Exonic single-nucleotide variants (SNVs) are generally classified as missense or nonsense. However, recent studies have revealed that exonic variants may cause aberrant splicing, even when they appear to be synonymous variants [12–16].

The splicing of nuclear precursor messenger RNA (pre-mRNA) is an essential step in gene expression, carried out by sophisticated ribonucleoproteins called spliceosomes. The spliceosome recognizes splicing signals and catalyzes the removal of non-coding intronic sequences from pre-mRNA, leading to the assembly of protein-coding sequences into mature mRNA [17–20]. Splicing signals are sequence-specific elements located at exon–intron boundaries (splice sites), the polypyrimidine tract, and the branch point [20]. The 5′ splice site is characterized by a consensus sequence, which includes the last three bases of exons and the first six nucleotides of introns—MAG/GURAGU at the exon/intron junction (M is adenine or cytosine, r is cytosine or thymine) [20–22]. However, compared to intronic variants, the possible roles of the exonic SNVs located near exon–intron boundaries in mediating splicing defects tend to be overlooked. We previously investigated the splicing effect of SNVs at the last nucleotide of exons in *COL4A5*, which revealed that as many as 85% of reported variants caused aberrant splicing [9]. Therefore, we hypothesized that SNVs positioned 2nd or 3rd to the last nucleotide of exons may also affect splicing. The aim of this study was to evaluate the splicing effect of exonic variants in *COL4A5*, positioned 2nd or 3rd to the last nucleotide of each exon.

## Materials and methods

### Variant nomenclature

The variant nomenclature followed the guidelines specified by the Human Genome Variation Society (http://varnomen.hgvs.org) using the NCBI Reference Sequence NM_000495.5.

### Candidate variants

**Fig. 1:**
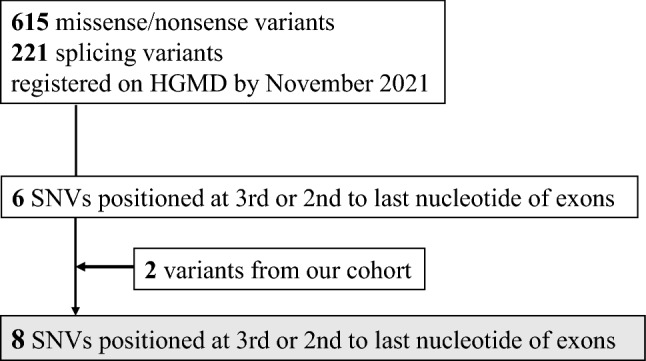
Selection of the candidate variants for this study. From the disease database, Human Gene Variant Database Professional (HGMD), six exonic single nucleotide variants (SNVs) positioned 2nd or 3rd to the last nucleotide in *COL4A5* were selected for this study. Two novel variants from our cohort were also included. Thus, a total of eight variants were analyzed

Candidate variants for this study were selected from the Human Gene Variant Database Professional (HGMD; accessed on November 2021). Among the 615 missense/nonsense variants and a total of 221 splicing variants registered on HGMD as disease-causing, all six SNVs positioned 2nd or 3rd to the last nucleotide of exons were selected for this study. Two novel variants from our cohort were also included; thus, eight variants were analyzed (Fig. 1).

### Genetic analysis with next-generation sequencing

Two novel variants were detected using next-generation sequencing (NGS). Genomic DNA was extracted from peripheral blood leukocytes obtained from patients and their family members using the Quick Gene Mini 80 system (Wako Pure Chemical Industries, Tokyo, Japan). Library preparation for NGS was conducted using the HaloPlex Target Enrichment System Kit (Agilent Technologies, Santa Clara, CA, USA) in accordance with the manufacturer’s instructions and then subjected to the MiSeq platform (Illumina, San Diego, CA). Sequenced data were aligned to the reference human genome (GRCh37/Hg19) and analyzed with SureCall 4.0, a desktop application combining algorithms for end-to-end NGS data analysis, from alignment to categorization of variants (Agilent Technologies).

### Bioinformatic analysis and interpretation of pathogenicity of the variants

We used the computational prediction software SIFT (https://sift.bii.astar.edu.sg/), PolyPhen-2 (http://genetics.bwh.harvard.edu/pph2/), Variant Taster (http://www.varianttaster.org/), and CADD (https://cadd.gs.washington.edu/snv) to classify the variants as pathogenic, likely pathogenic, or uncertain significance, according to the guidelines of the American College of Medical Genetics and Genomics (ACMG) [23]^.^ To predict the strength of the splice sites, we used SpliceSiteFinder-like (http://www.interactive-biosoftware.com), MaxEntScan (http://hollywood.mit.edu/burgelab/maxent/Xmaxentscan_scoreseq.html), and NNSplice via the Alamut software v.2.11 (Interactive Biosftware, Rouen, France; http://www.interactive-biosoftware.com) with default settings. Each tool was estimated to predict altered splicing when the change in splice site score was ≥ 10% (MaxEntScan) or ≥ 5% (SpliceSiteFinder-like and NNSplice) [24, 25]. Additionally, SpliceAI, a deep learning-based tool to identify splice variants on a web-based interface (https://spliceailookup.broadinstitute.org/), was used to assess the potential of splicing defects in the variants using a Δ Score > 0.2 as the cutoff [26].

### Reverse transcription-polymerase chain reaction (RT-PCR) analysis for mRNA obtained from patient blood samples

For variants 2, 4 and 8, cDNA analysis was performed as patient blood samples were available. Total RNA was extracted from blood leukocytes using the RiboPure Blood Kit (Invitrogen, Carlsbad, CA, USA) and an RNA stabilization agent (RNAlater, Invitrogen). Total RNA of 2 µg was converted to cDNA via reverse transcription with EcoDry Premix (Double Primed; Takara Bio Inc., Tokyo, Japan), and analyzed by PCR amplification.

### X-chromosome inactivation analysis

As for variants 2 and 8, the samples used in the cDNA analysis were obtained from female patients. Thus, we performed methylation-based X-chromosome inactivation analysis to interpret the results of the cDNA analysis. Genomic DNA (200 ng) was incubated overnight at 37 °C with or without 25 units of Hpa II (New England BioLabs, Inc., Ipswich, MA) in a total volume of 20 µL. After deactivating enzymes at 80 °C for 30 min, the digested DNA was used to amplify the segment spanning the CAG repeats in the human androgen receptor gene (HUMARA) and the extragenic GAAA repeats of the retinitis pigmentosa-2 (RP2) gene, respectively. Skewing was defined as an XCI ratio ≧3:1. This definition has been used in previous studies [27].

### Minigene splicing assay

The hybrid minigene splicing assay was constructed using the H492 vector, based on the mammalian expression vector pcDNA 3.0 (Invitrogen; Supplementary Fig. S1). Genomic DNA samples from healthy controls and patients were amplified to obtain DNA fragments, including exons and approximately 200 bp of introns adjacent to each targeted exon. The H492 vector was linearized either by PCR or with restriction enzymes (*NheI* and *BamHI*), and amplicons were subsequently cloned into the vector via in-fusion cloning to construct wild-type or mutant plasmids. If patient samples were unavailable, site-directed mutagenesis was performed using the PrimeSTAR Mutagenesis Basal Kit (Takara Bio Inc.). Each minigene was transfected into HEK293T cells using Lipofectamine 2000 (Invitrogen), following the manufacturer’s instructions. The cells were then incubated for 24 h, total RNA was extracted using the RNeasy Plus Mini Kit (QIAGEN, GmbH, Hilden, Germany), and it was then reverse-transcribed using EcoDry Premix (Double Primed; Takara Bio Inc.). RT-PCR was conducted with primers YH307 and YH308, which are homologous to H492 exons A and B, respectively. Amplicons were analyzed by electrophoresis on a 1.5% agarose gel using the ϕX174-Hae III digest marker and direct sequencing. The sequences of primers used for the minigene splicing assay are described in Supplementary Table S1. Abnormal splicing was determined if any of the following criteria were met: (i) WT with only normal splicing and the variant with only aberrant splicing; (ii) WT with only normal splicing and the variant with both normal and aberrant splicing; or (iii) WT with both normal and aberrant splicing and the variant with only aberrant splicing.

## Results

**Fig. 2:**
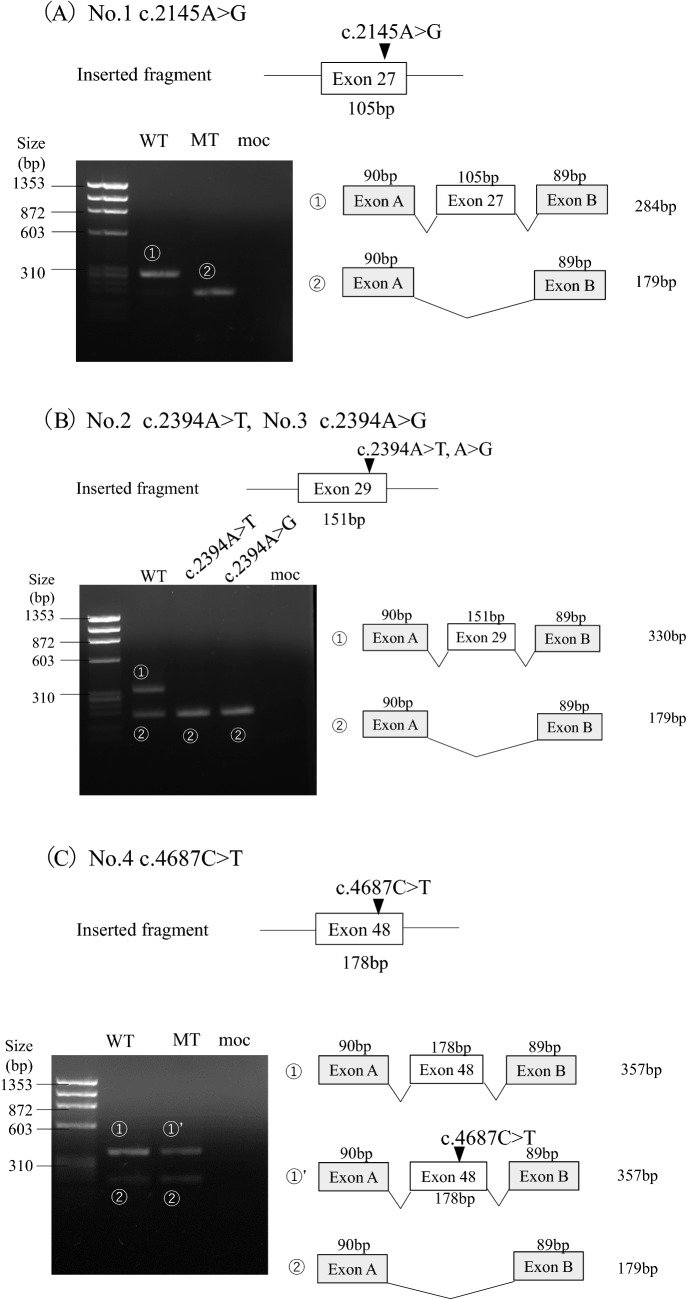

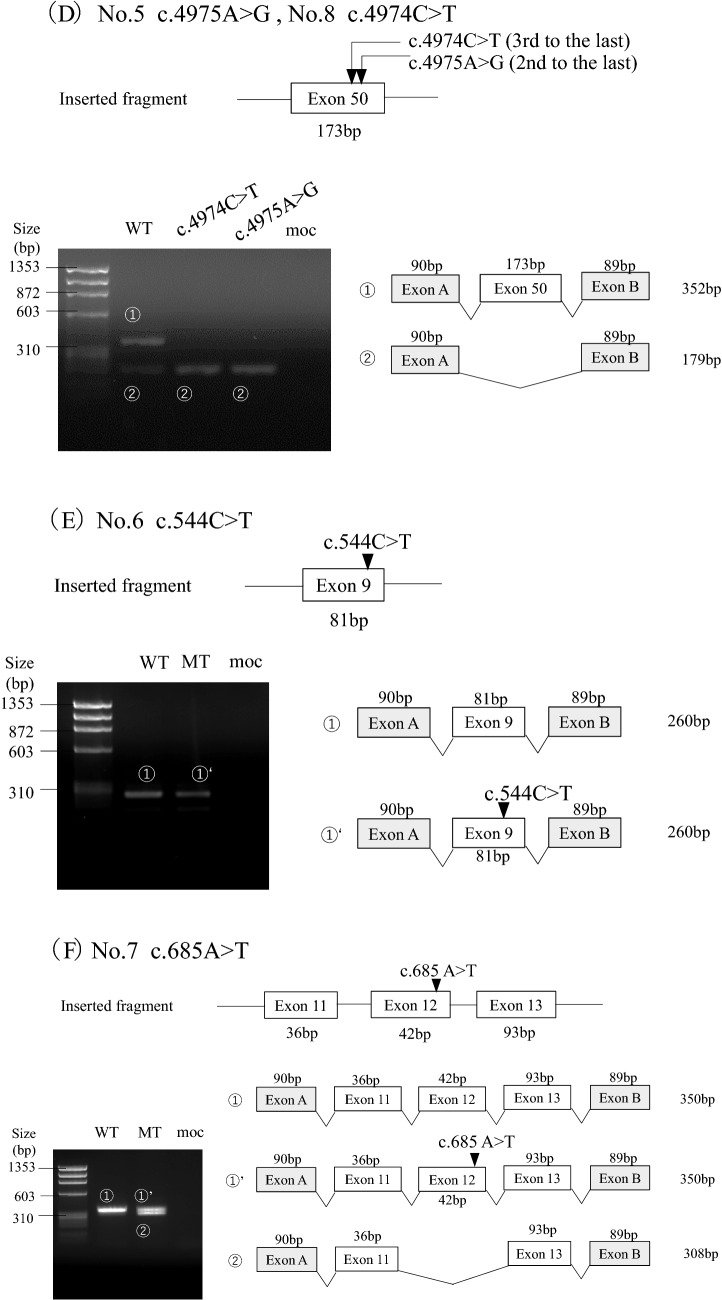
Transcriptional analysis of splicing reporter minigene assay for the variants. The upper part of the figure shows each inserted fragment constructed with individual exons and flanking introns. On the lower left, agarose gel electrophoresis of the RT-PCR product of minigene transcripts in HEK293T cells is shown. The sizes of the DNA marker are indicated to the left of each image. A schematic representation of the splicing outcome is shown on the lower right. Exons are represented by boxes. All PCR products were verified by sequencing. **A** Variant 1 c.2145A > G showed exon 27 skipping. **B** Both variants 2 c.2394A > T and 3 c.2394A > G resulted in exon 29 skipping. **C** Variant 4 c.4687C > T caused normal splicing. **D** Both variants 5 c.4975A > G and 8 c.4974C > T caused exon 50 skipping. **E** Variant 6 c.544C > T produced exon 9 skipping. **F** Variant 7 c.685A > T resulted in both exon 12 skipping and normal splicing

Three candidate variants were identified in the 3rd to the last nucleotide of the exon, whereas the others were located in the 2nd nucleotide (Table 1). To examine the genotype–phenotype correlation, clinical data from male patients only are included in Table 1. According to the ACMG guidelines, three variants (4, 6, and 7) were determined as pathogenic; however, we were uncertain about the others (Supplementary Table S2). The initial classification of the candidate variants was as follows: three nonsense, two missense, and three synonymous variants (Table 1). The minigene splicing assay revealed that exon skipping occurred in six variants (1, 2, 3, 5, 7 and 8; Table 1; Fig. 2). Among them, four variants, initially assessed as non-truncating, were revealed to be truncating transcripts (variants 2, 3, 5 and 8). Variant 7, initially assessed as a nonsense variant, generated both exon 12 skipping and non-skipping transcripts. The remaining variants 4 and 6, whose initial classification was nonsense, showed normal splicing. Variants 2, 4 and 8 were subjected to RT-PCR for patients’ mRNA, and the results corroborated those of the minigene splicing assay (Supplementary Fig. S2). Variants 2 (A864.1) and 8 (A516.2) were from female patients. In general, transcript analysis of heterozygous female patients shows not only transcripts resulting from the mutant allele but also transcripts from the unaffected allele. If a normally spliced transcript does not carry the variant, the aberrantly spliced transcript is considered to be generated from a mutant allele and we can infer that the variant produced only aberrantly spliced transcripts. Patient A864.1 was a 5-year-old girl with overt proteinuria (1.5 g/gCr) and preserved kidney function (eGFR: 153 mL/min/1.73 m^2^). At the age of 3 years, she was diagnosed to have hematuria and proteinuria, which worsened after a streptococcal infection contracted at the age of 4 years. Patient A516.2 was a 3-year-old girl who presented with hematuria and proteinuria (0.95 g/gCr). Her kidney function was preserved (eGFR: 152 mL/min/1.73 m^2^). She was noted to have hematuria and occasional macrohematuria at the age of 3 years.

**Table 1:** Clinical information and splicing outcome of the candidate variants

Variant No	Variant			Splicing outcome				Prediction of Splice Site Score			SpliceAI	Clinical information (male patients)						
								SSF-like	MaxEntScan	Nnsplice	Δscore	ID/Ref		Ageat analysis	Age detected UP	Age developed To ESKD	Ocular abnormalities	Hearing loss
	Exon (bp)	Nucleotide	Protein	cDNA analysis (ID/Gender)	Minigene splicing reporter assay		Confirmed variant type	Score change (%)	Score change (%)	score change (%)								
					Splicing outcome	Protein effect												
2nd to the last nucleotide of exon																		
1	27 (105)	c.2145A > G	p.Lys715 =	ND	exon27 skipping	p.Asp682_Gly716del	in-frame del	**− 9.9**	**− 15.3**	**− 10**	0.05	Ref.36^#^		ND	ND	ND	ND	ND
2	29 (151)	c.2394A > T	p.Lys798Asn	exon29 skipping (A864.1/ F)	exon29 skipping	p.Gly749Valfs*20	out-frame del	**− 10.1**	**− 34.4**	**− 10**	**0.57**	A864.1^※^		ND	ND	ND	ND	ND
3		c.2394A > G	p.Lys798 =	ND	exon29 skipping	p.Gly749Valfs*20	out-frame del	**− 10.6**	**− 24.7**	**− 10**	**0.71**	Ref.6^#^		ND	ND	ND	ND	ND
4	48 (178)	c.4687C > T	p.Arg1563*	Normal splicing (A880.1/M)	Normal splicing	p. Arg1563*	nonsense	0.5	**− 15.2**	0	0.18	A75.1		16 y	ND	ND	–	–
												A440.1		1 y	3 m	–	ND	–
												A880.1		2 y	1 y 3 m	–	–	–
												Ref 37	1	ND	2 y	23 y	–	11 y
													2	ND	5 y	ND	–	+ (early age)
													3	ND	8 y	29 y	+	7 y
5	50 (173)	c.4975A > G	p.Ser1659Gly	ND	exon50 skipping	p.His1602*	nonsense	**− 10.7**	**− 36.1**	**− 44.4**	**0.58**	Ref.38^#^		ND	ND	ND	ND	ND
3rd to the last nucleotide of exon																		
6	9 (81)	c.544C > T	p.Gln182*	ND	Normal splicing	p.Gln182*	nonsense	− 4.8	**− 19.8**	0	0.03	Ref.39^#^		ND	ND	ND	ND	ND
7	12 (42)	c.685A > T	p.Lys229*	ND	Normal splicing	p.Lys229*	nonsense	− 4.6	− **23.8**	**− 10**	**0.29**	Ref.40		26	ND	-	ND	+
					exon12 skipping	p.Asn217_Gly230del	in-frame del											
8	50 (173)	c.4974C > T	p.Phe1658 =	exon50 skipping (A516.2/ F)	exon50 skipping	p.His1602*	nonsense	**− 5**	− **28.9**	**− 44.4**	**0.37**	A516.1		ND	2 y	15–20 y	ND	+

Bold figures met the prediction criteria described in the Methods section

*HGMD* Human Gene Variant Database Professional, *UP* urinary protein, *ESKD* end-stage kidney disease, *del* deletion, *SSF-like* SpliceSiteFinder-like, *MES* MaxEntScan, *HSF* Human Splicing Finder, *ND* no data, *M* male, *f* female, *y* year, *Ref* reference

^※^There was no affected male patient in the family

^#^No detailed clinical information for the respective male patient. -not developed ESKD, not having ocular abnormalities or hearing loss

X chromosome inactivation analysis revealed that A864.1 showed a skewed X inactivation pattern, whereas A516.2 showed a random X inactivation pattern (Supplementary Fig. S3).

Each sequence of the RT-PCR products generated from the minigene assay is shown in Supplementary Fig. S4. *In-silico* prediction of the strength of the splice site using the Alamut program correctly predicted all six aberrant splice sites (Table 1; bold figures met the prediction criteria described in the Methods section). SpliceAI correctly predicted five splicing defects (Table 1).

## Discussion

In this study, we revealed that six out of eight exonic SNVs positioned 2nd or 3rd to the last nucleotide of exons in *COL4A5* caused aberrant splicing. We previously reported that 17 out of 20 SNVs at the end of exons in *COL4A5* affected splicing [9]; therefore, 23 out of 28 (82%) disease-causing SNVs of the three terminal nucleotides of exons cause splicing defects. To date, 30,191 (8.6%) out of 35,2731 disease-causing variants have been assigned as splicing variants in HGMD (released in 2021.4). However, the reported number of splicing variants is likely underestimated. Sterne-Weiler et al. reported that up to 25% of known missense and nonsense disease-causing variants alter the functional splicing signals within exons [28]. The results from our study strongly suggest that care has to be taken when interpreting the pathogenicity of exonic SNVs near the 5′ splice site.

The results of the cDNA analysis for variants 2, 4, and 8, were consistent with those of the minigene assay. For variants 2 (A864.1) and 8 (A516.2), we performed methylation-based X-chromosome inactivation analysis to interpret the results of the cDNA analysis because the samples used in the cDNA analysis were obtained from female patients. A864.1 showed a skewed X inactivation pattern, whereas A516.2 showed a random X inactivation pattern. Since the parents of A864.1 did not have the variant and it was a *de-novo* variant, it was impossible to determine whether the higher activated allele had the variant or not. However, cDNA analysis showed that despite the lower amplification efficiency, the normally spliced longer transcripts were amplified more than the shorter transcripts generated from the mutant alleles, which probably resulted from the skewed X chromosome inactivation. On the other hand, even though A516.2 showed a random X inactivation pattern, cDNA analysis showed only exon-skipped transcripts. It often occurs that only one transcript is detectable when using cDNA obtained from female patients, which results from the differences in PCR efficacy. Therefore, we detected only a short transcript generated from the mutant allele of patient A516.2.

The recent progress in high-throughput sequencing technologies have generated *in-silico* prediction tools. In our study, six variants (1, 2, 3, 5, 7 and 8) met the criteria of the altered splicing score described in the Methods, resulting in all six variants causing aberrant splicing. SpliceAI accurately predicted aberrant splicing in five of these variants (2, 3, 5, 7, and 8), but could not predict the alternative splicing of variant 4 (synonymous variant). Although SpliceAI reportedly outperforms other *in-silico* prediction tools [29, 30], Riepe et al*.* reported that SpliceSiteFinder-like was found to perform better on near-splice site variants [31]. Thus, we propose that it would be better to combine these tools to predict alternative splicing precisely.

Alternative splicing is classified into five types: intron retention, alternative 5′ splice site, alternative 3′ splice site, exon skipping, and mutually exclusive exons (in which only one of two or more candidate exons is spliced into the mature mRNA isoform) [32, 33]. Kurmangaliyev et al*.* comprehensively analyzed splicing variants and compared the characteristics of skipped exons (S-exons) and exons utilizing cryptic sites (C-exons) [34]. They reported that S-exons were significantly shorter than C-exons (median lengths were 114 nt and 136 nt, respectively), but there was no significant difference in the scores of the authentic 5′ splice sites between S-exons and C-exons [34]. In our recent study, all six variants resulting in splicing alteration showed exon skipping, despite no correlation between exon length and splicing. Although *in-silico* tools are reliable in predicting the possibility of aberrant splicing, it is impossible to thoroughly evaluate the type of alternative splicing caused by the identified variants.

Several studies have revealed that male patients with XLAS show a straightforward genotype–phenotype correlation. Bekheirnia et al. reported that the average age at onset of end-stage kidney disease (ESKD) in XLAS males was 37 years for those with missense variants, 28 years for those with splice-site variants and 25 years for those with truncating variants [6]. In addition, we previously reported that XLAS patients harboring truncating splicing abnormalities have significantly poorer renal prognoses than those with non-truncating splicing abnormalities [10]. In this study, the minigene splicing assay revealed that variant 7 (initially assessed as a nonsense variant) generated both exon 12 skipping and non-skipping transcripts. Exon 12 consists of 42 bp (multiples of 3) nucleotides; thus exon 12 skipping results in an in-frame deletion. It is well known that male XLAS patients harboring in-frame deletions reach ESRD later than those with frameshift variants [5]. According to a report on variant 7, a male patient showed a relatively mild phenotype, not having developed ESKD at the age of 26. This suggests that splicing defects cause in-frame deletions in a certain part of transcripts, which may reduce the severity of the disease. For variant 8, initially classified as a synonymous variant, both cDNA analysis and the minigene splicing assay showed exon 50 skipping, which resulted in a truncating variant. Male patients harboring this variant develop ESKD between 15 and 20 years of age, which is consistent with the cDNA analysis and minigene assay results. Thus, it is critical to determine whether these variants result in truncated or non-truncated transcripts to predict kidney prognosis.

A recent innovation called “exon skipping therapy” was developed using single-stranded antisense oligonucleotides (ASOs). In XLAS, ASO therapy targeting exon 21 in *COL4A5* significantly improved the clinical phenotypes of a mouse model of AS, suggesting that exon skipping may represent a promising therapeutic approach for treating severe male XLAS cases [35]. Thus, understanding the impact on transcripts generated from identified variants is becoming more clinically essential. In this context, the minigene splicing assay helps confirm the effect of variants on splicing, especially for genes with a genotype–phenotype correlation similar to that of *COL4A5*.

Our study had some limitations. First, the number of mutations verified in this study was limited because there were a total of six eligible mutations, even though all variants registered in the HGMD were tallied. Therefore, further investigations targeting other exons of *COL4A5* or even other COL4A genes should be continuously examined. Second, limited clinical information is available for some reported variants. Therefore, the association between splicing abnormalities and the clinical course has not been fully verified. Third, we could not perform an RT-PCR analysis of mRNA obtained from patients for all variants, as most variants were not included in our cohort.

## Conclusion

We revealed that exonic SNVs positioned 2nd or 3rd to the last nucleotide of the exon in *COL4A5* could cause aberrant splicing. The results of this study suggest that we should pay attention when interpreting the pathogenicity of exonic SNVs near the 5′ splice site.

## Supplementary Information

Below is the link to the electronic supplementary material.

Supplementary file1 (DOCX 2417 KB)

## Data Availability

The data are not publicly accessible because of patient privacy concerns but are available from the corresponding author upon reasonable request.
