# Supplementary material for: Aberrant splicing caused by exonic single nucleotide variants positioned 2nd or 3rd to the last nucleotide in the COL4A5 gene
Source: Clin Exp Nephrol. 2022 Nov 12;27(3):218–26. doi: 10.1007/s10157-022-02294-x (PMC9950164; doi:10.1007/s10157-022-02294-x)
Supplement: Supplementary file 1 — Supplementary file1 (DOCX 2417 KB) [file 10157_2022_2294_MOESM1_ESM.docx]

Supplementary materials

**Aberrant splicing caused by Exonic Single Nucleotide Variants Positioned at 2nd and 3rd to the Last Nucleotide in COL4A5 Gene**

Contents

**Supplementary Figure S1.** Schematic representation of the H492 vector

**Supplementary Table S1.** Primer sequences

**Supplementary Table S2.** Bioinformatic analysis and interpretation of pathogenicity of the variants

**Supplementary Figure S2.** Transcript analysis of cDNA obtained from peripheral blood leukocytes

of the patients (A864.1, A880.1 and A516.2)

**Supplementary Figure S3.** X-chromosome inactivation analysis of A864.1 and A516.2

**Supplementary Figure S4.** Sequences of RT-PCR product generated from minigene assay

**Supplementary Figure S1.** Schematic representation of the H492 vector


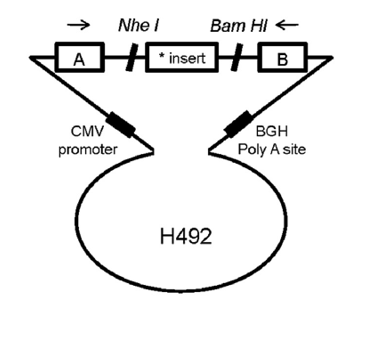


**Supplementary Table S1.** Primer sequences

**Supplementary Table S2.** Bioinformatic analysis and interpretation of pathogenicity of the variants

HGMD, Human Gene Mutation Database Professional; ACMG, American College of Medical Genetics and Genomics.

**Supplementary Figure S2.** Transcript analysis of cDNA obtained from peripheral blood leukocytes of the patients

1. No.2 c.2394A>T (A864.1, female patient)

**
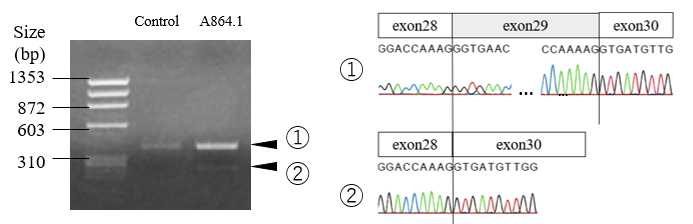
**

**(B)**No.4 c.4687C>T (A880.1, male patient)


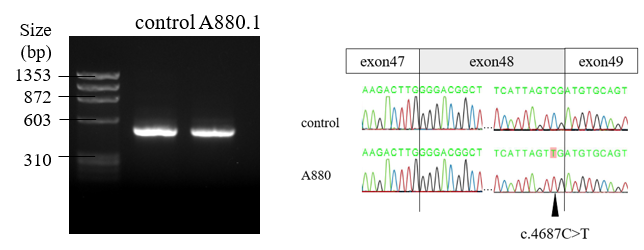


**(C)** No.8 c.4974C>T (A516.2, female patient)

**
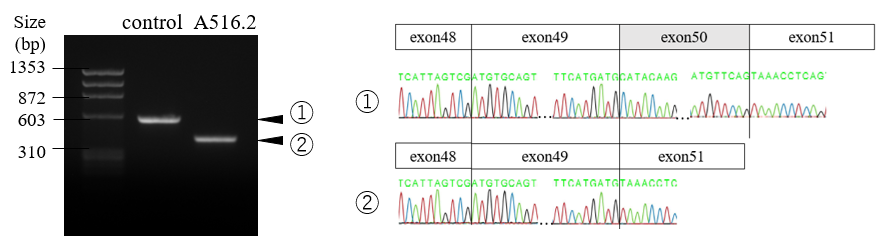
**

**Supplementary Figure S3.** X-chromosome inactivation analysis of A864.1 and A516.2

**（A）**No.2 c.2394A>T (A864.1, female patient)

**
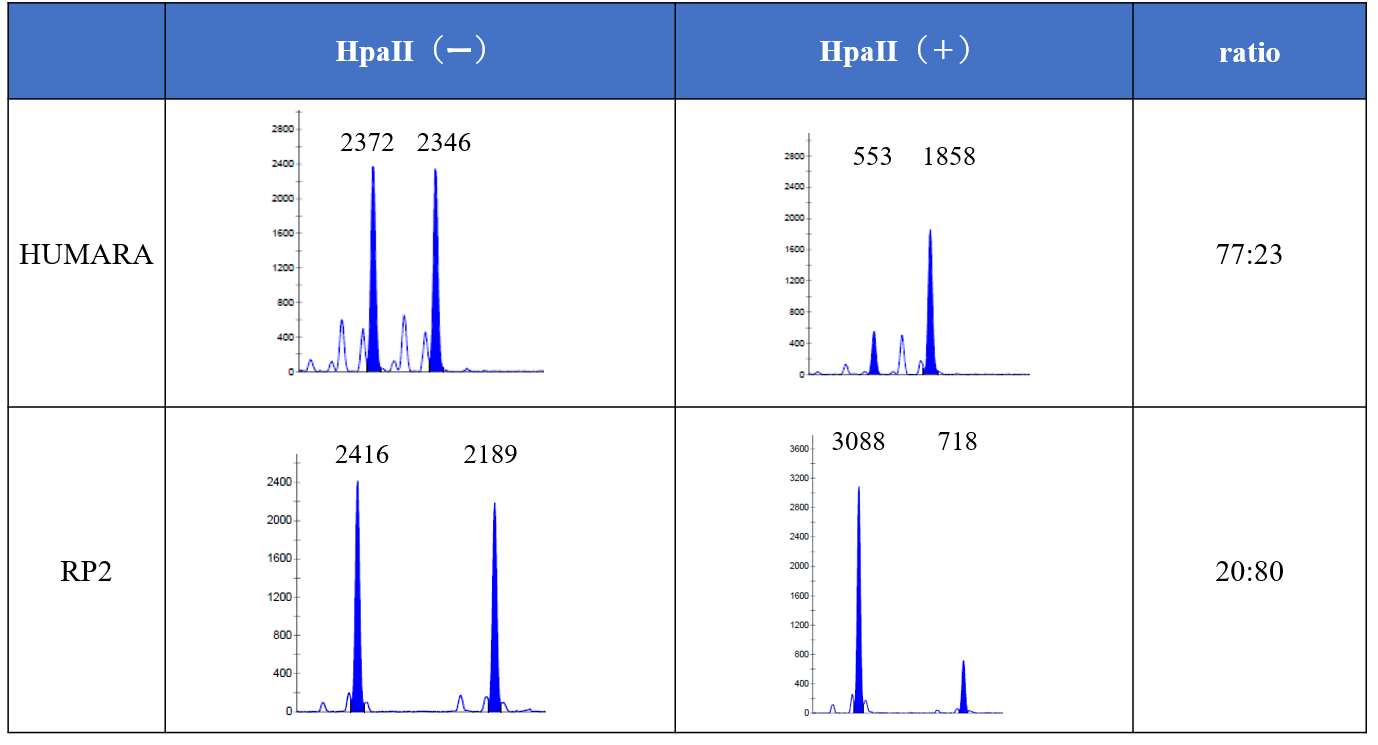
**

**（B）** No.8 c.4974C>T (A516.2, female patient)

**
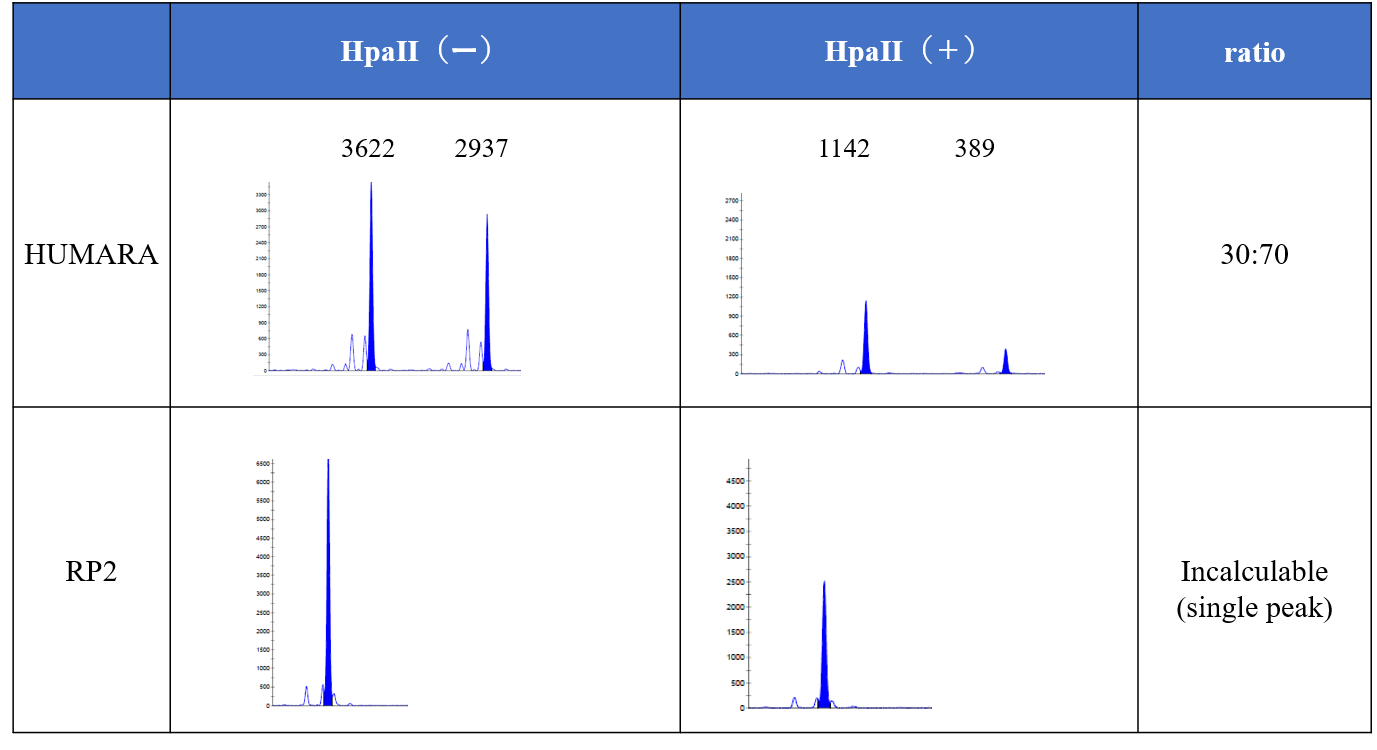
**

**Supplementary Figure S4.** Sequences of RT-PCR product generated from minigene assay

**（A）**No.1 c.2145A>G

①WT


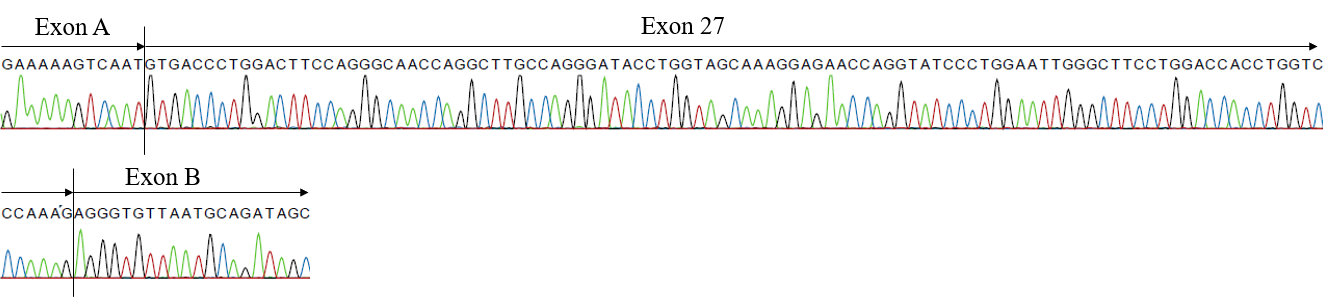


1. MT


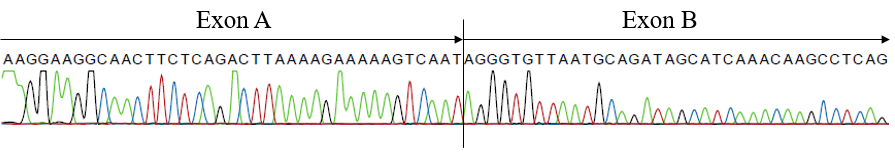


**（B）**No.2 c.2394A>T, No.3 c.2394A>G

1. WT


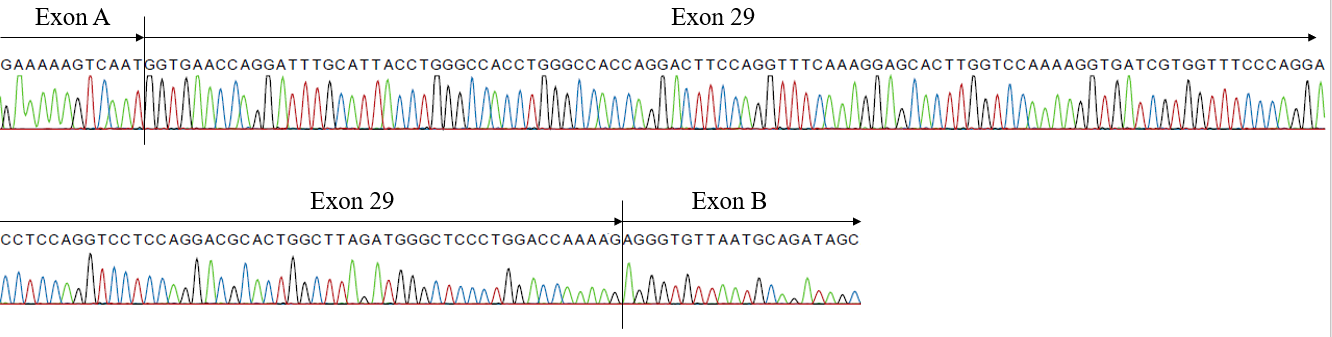


1. WT, MT (c.2394A>T, c.2394A>G)


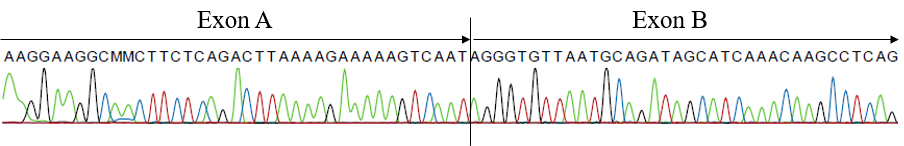


**(C)** No.4 c.4687C>T

①　WT


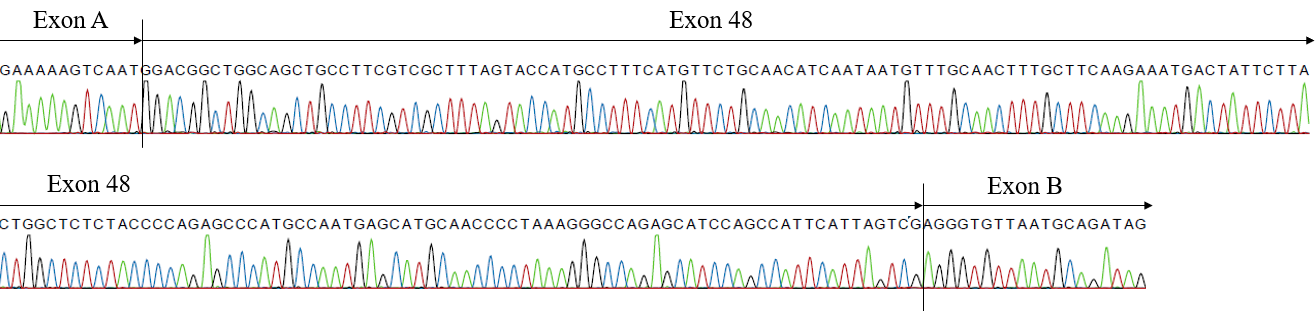


1. ′　MT（c.4687C>T）


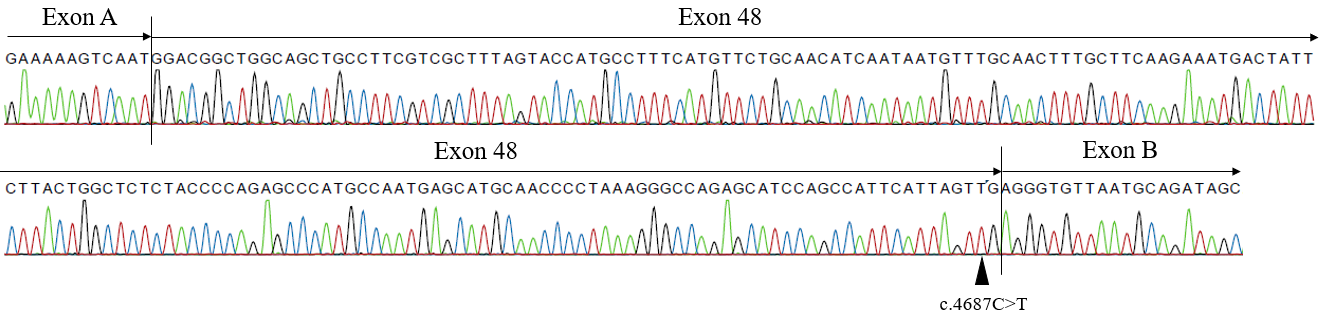


1. WT, MT（c.4687C>T）


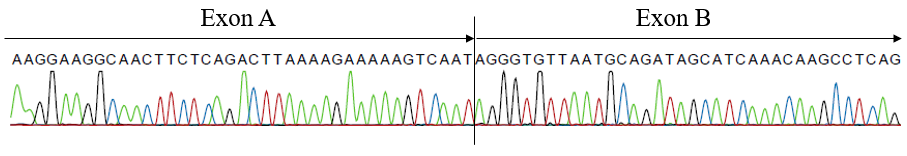


**(D)** No.5 c.4975A>G, No.8 c.4974C>T

①　WT


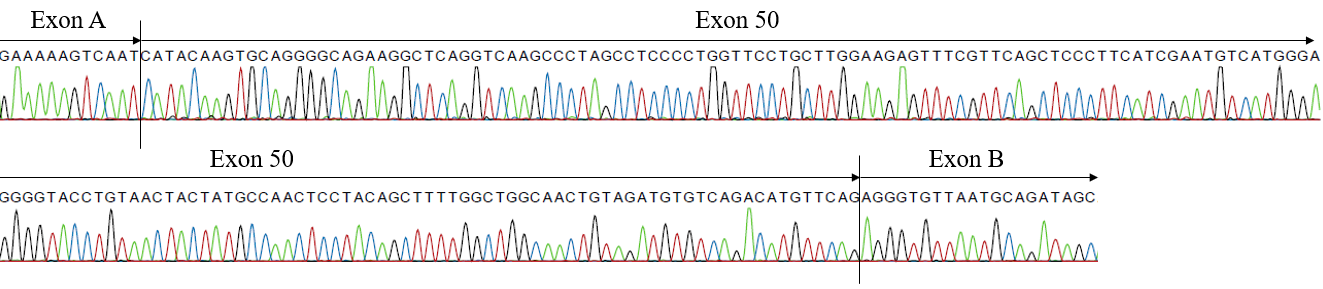


- 1. WT, MT（c.4974C>T, c.4975A>G）


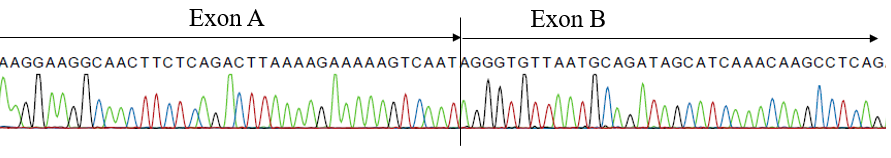


**(E)** No.6 c.544C>T

1. WT


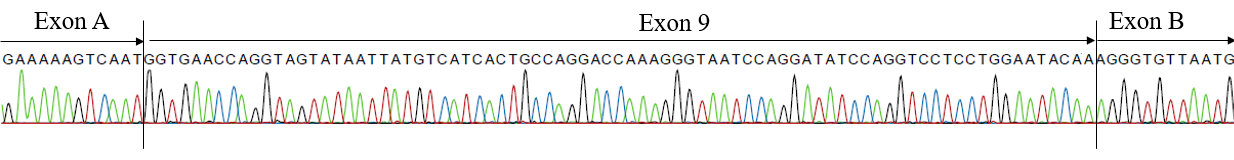


- 1. ′MT (c.544C>T)


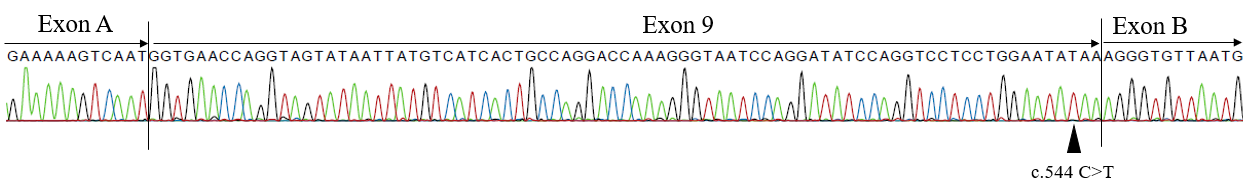


**(F)** No.7 c.685A>T

1. WT


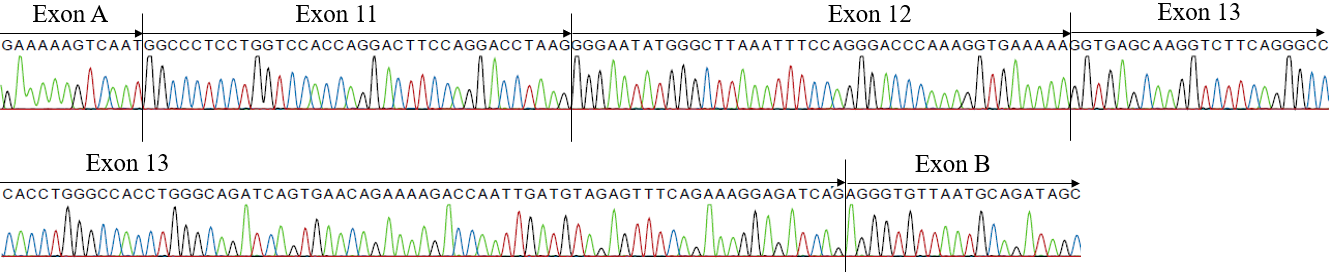


①′ MT (c.685A>T)


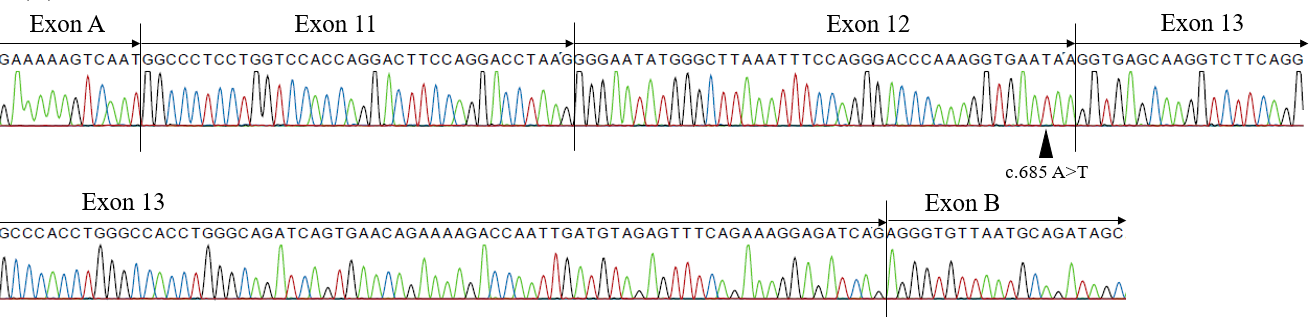


1. MT (c.685A>T)


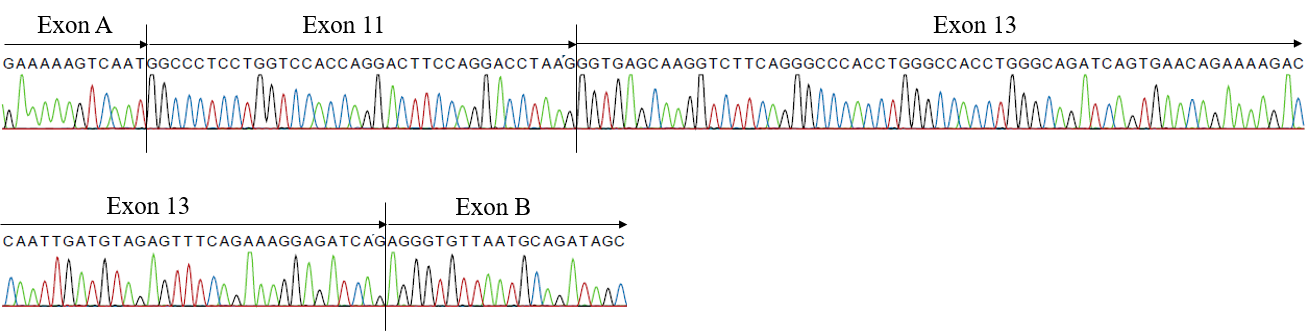


**Supplementary Figure Legends**

**Supplementary Figure S1.**

The H492 vector contains two cassette exons, A and B, separated by their natural intron into which we have inserted appropriate cloning sites. The H492 vector also contains a cytomegalovirus (CMV) enhancer-promotor and a bovine growth hormone gene (BGH) polyadenylation site.

**Supplementary Figure S2.**

The images on the left show the electropherogram and the results of direct sequence of each PCR product are exhibited on the right. **(A)** The female patient (A864.1) with the No.2 variant c.2394A>T showed exon 48 skipped and normal splicing. In general, transcript analysis of heterozygous female patients shows not only transcript resulting from the mutant allele but also transcript from unaffected allele. **(B)** The male patient A880.1 harboring the No.4 c.4687C>T variant exhibited normal splicing. **(C)** The female patient (A516.2) harboring the No.8 c.4974C>T variant showed exon 50 skipping. Only mutated transcript was detectable, which may be caused by the difference of PCR efficacy of the transcripts.

**Supplementary Figure S3.**

Results of methylation-specific PCR assay for HUMARA and RP2 gene. Figures on the left show PCR assay results without HpaⅡdigestion and that on the right show results after HpaⅡdigestion. The activity ratios were described as shorter allele : longer allele. The results of A864.1 shows skewed X inactivation pattern **(A)**, whereas the results of A516.2 shows random X inactivation pattern **(B)**.

**Supplementary Figure S4.**

**(A)** WT transcript exhibited exon 27 inclusion (①), while MT transcript lacked exon 27 sequence (②). **(B)** Transcript generated from WT contains exon 29 sequence (①), whereas transcript obtained from MT showed exon 29 skipping (②). **(C)** Both WT and MT showed 2 types of transcript : one is containing exon 48 (①, ①′) and the one without exon 48 (②).

**(D)** Transcript resulting from WT contains exon 50 sequence (①), whereas transcript obtained from MT showed exon 50 skipping (②). **(E)** Both WT (①) and MT (①′) contained whole sequence of exon 9. **(F)** Transcript generated from WT plasmid contained exon 11, 12 and 13 (①). MT exhibited 2 transcripts: including exon 11, 12 and 13 (①′) and exon 12 skipped (②).
